# Hearing movement, seeing sound: multimodal predictive coding in pianist-dancer interaction

**DOI:** 10.3389/fpsyg.2026.1820643

**Published:** 2026-05-08

**Authors:** Xinyu Cao, Xinlei Shi

**Affiliations:** Beihua University, Jilin, China

**Keywords:** active inference, coupled oscillator model, EEG hyperscanning, joint action, micro-timing perturbation, pianist–dancer interaction, predictive coding, sensorimotor synchronization

## Abstract

Live piano accompaniment for dance poses a “zero-latency paradox”: performers achieve near-simultaneous audiovisual alignment despite sensory and integration delays that should make purely reactive control too slow. This review argues that pianist–dancer coordination can be usefully framed as bidirectional inference under cross-modal predictive coding, supported by converging behavioral, kinematic, and neurophysiological evidence. Motion-capture and time-series studies suggest that pianists can use dancers' preparatory kinematics, such as trajectory shifts and acceleration changes, to shorten the prediction window for timing and dynamics, while neuroimaging and EEG findings indicate action–perception coupling consistent with internal simulation of action–sound mappings (“seeing sound”). Sensorimotor synchronization paradigms show that micro-timing perturbations in auditory beats elicit rapid, asymmetric phase correction in stepping and tapping, consistent with predictive control in dancers (“hearing movement”), and autonomic measures further suggest that musical tension can modulate arousal before overt movement changes. Integrating coupled-oscillator modeling and EEG hyperscanning, we highlight quantifiable bidirectional adaptation and converging evidence that coordination is dynamically co-regulated rather than purely reactive or unidirectional. Taken together, the reviewed literature supports a neurally informed account of closed-loop dyadic coordination while also underscoring the need for more direct evidence from pianist–dancer interaction itself.

## 1 Introduction

In live dance accompaniment, pianists often introduce changes in beat and dynamics before a dancer raises an arm, lands, or changes direction, creating the impression of zero latency. However, if coordination relied only on visual or auditory feedback, neural transmission and integration delays—often on the order of tens to hundreds of milliseconds—would not support such high-precision synchrony. In professional performance, *perfect* synchrony is sometimes believed (based on experience) to involve a shared target timing error within approximately ±5 ms, implying that performers must initiate actions before they can hear their partners' sounds (Akao, 2025). This constitutes the “zero-latency paradox”: synchrony is not one-way reaction, but real-time inference by both partners based on incomplete cues. Recent evidence supports a cue-to-prediction account. Sensorimotor synchronization is typically less stable for visual than for auditory/tactile timing cues (Whitton and Jiang, 2023), and dancers select specific movement reference points as anchors that bind action to the beat (Wakiyama et al., 2025). Taken together, these findings motivate an inference-based account of coordination and make predictive coding a useful framework for organizing the available evidence. Music processing relies on continuous prediction and updates generative models *via* prediction error (Vuust et al., 2022), and bodily engagement can change the precision-weighting of rhythmic prediction errors (Ishida and Nittono, 2025). This review synthesizes recent empirical studies, supplemented by foundational work in synchronization, action observation, and joint action, to examine how pianists and dancers use multimodal micro-cues to construct shared generative models that support cross-modal anticipatory timing and rapid online micro-adjustments. It also discusses implications for training and research design within a joint musical coordination framework (Abalde et al., 2024).

## 2 Theoretical framework: predictive coding as an integrative account of cross-modal joint action

Several theoretical perspectives can inform pianist–dancer coordination, including dynamical-systems/entrainment accounts, ecological approaches centered on perception–action coupling, and broader joint-action frameworks emphasizing shared task structure and mutual adaptation (Abalde et al., 2024; De Vicariis et al., 2024; Heggli et al., 2019, 2021; Phillips-Silver et al., 2010; Warren, 1990). We do not treat predictive coding as displacing these alternatives. Rather, we adopt it here as an integrative framework because it can accommodate anticipatory cue use, probabilistic expectation, precision-weighted error correction, and bidirectional online adaptation within a single explanatory vocabulary (Abalde et al., 2024; Vuust et al., 2022). In this sense, coupled-oscillator and synchronization models are treated not as competing accounts to be dismissed, but as formal descriptions of coordination dynamics that can be interpreted within a predictive, inference-based framework (De Vicariis et al., 2024; Heggli et al., 2019, 2021).

In pianist–dancer joint action, the core assumption of cross-modal predictive coding is that performers do not wait for external input and then react. Instead, each maintains a hierarchical generative model that continuously predicts the auditory, visual, and proprioceptive evidence likely to occur in the next moment, and prediction error drives rapid correction and strategy updating (Abalde et al., 2024; Vuust et al., 2022). Accordingly, shared generative models are not a static joint plan; they are probabilistic internal representations that gradually align through coupled interaction. Each performer predicts the sound consequences of their own actions and predicts how the partner's movement trajectory will reshape the temporal structure and expressive intent of the next beat (De Vicariis et al., 2024).

### 2.1 Generative models and priors

In predictive coding terms, priors are probabilistic expectations that the system brings to ongoing perception and action before incoming sensory evidence is fully resolved. In pianist–dancer coordination, such priors arise from two main sources. The first is rehearsal and experiential memory, including the breath–takeoff–landing rhythm in fixed passages, habitual rubato, and partner-specific statistics about where exaggerated preparatory actions tend to occur. The second is the statistical regularities of musical styles, such as metrical hierarchies, phrase boundaries, and high-probability locations of harmonic or rhythmic turning points. Recent neural-encoding work shows that musical expectations can be formalized with indices such as surprisal and entropy, and these indices explain gains in EEG-based reconstruction of melodic information, suggesting that the prior–uncertainty relation is not merely metaphorical but can be treated as a quantifiable predictive variable (Galeano-Otálvaro et al., 2024). In the present context, this implies that dancers' visual micro-cues (e.g., preparatory posture, weight shift) and pianists' auditory-structural cues (e.g., anacrusis, sustain, and touch intensity) can be incorporated into a single generative model to yield cross-modal next-event predictions.

### 2.2 Precision-weighted prediction error

When input deviates from expectation, the system does not correct simply because the error is larger. Instead, it weights prediction error by precision, that is, the estimated reliability or confidence of the cue: errors from reliable channels are amplified, and errors from unreliable channels are attenuated, reducing overall uncertainty (Limanowski et al., 2024). In performance, precision can be understood as dynamic weight allocation and attentional gain. For example, when complex syncopation or dancer occlusion reduces auditory predictability, the pianist may up-weight the precision of visual kinematics. Conversely, under stable beats and clear auditory boundaries, auditory prediction error is more likely to dominate micro-adjustments. Consistent with this account, active motor engagement (e.g., rhythmic tapping or body sway) can modulate prediction-error signals by increasing the certainty of beat information, reflecting active inference within an action–perception loop (Ishida and Nittono, 2025). In addition, canonical electrophysiological signatures of prediction error are substantially attention-dependent, making precision-as-attentional-gating empirically testable (Male and O'Shea, 2023).

Finally, in dyadic coordination, prediction is not only used to keep up with the partner; it is also used to make oneself predictable and legible. Performers can reduce the partner's inferential cost by exaggerating preparatory actions, breathing earlier, or stabilizing touch patterns, which supports more robust mutual correction and synchrony (Russo et al., 2025). We map priors, precision, error, and action onto observable multimodal micro-cues and timing-correction indices.

## 3 Seeing sound: empirical evidence for visual–acoustic prediction

Precise synchrony between pianists and dancers is unlikely to rely on auditory feedback alone; it plausibly benefits from anticipatory use of partners' visible kinematics. Because direct pianist–dancer evidence remains limited, the following subsections also draw on adjacent paradigms, including piano duets, ensemble cueing, musician action-observation, and dance-related observation studies. We treat these not as one-to-one substitutes for the pianist–dancer dyad, but as theoretically relevant analogs that share core coordination demands: temporally constrained interaction, partial access to a partner's evolving action, reliance on anticipatory multimodal cues, and rapid online adjustment. To the extent that these paradigms recruit comparable mechanisms of kinematic prediction, action–perception coupling, and error correction, they provide transferable evidence about how cross-modal inference may operate in pianist–dancer coordination, while still leaving the need for more direct dyadic evidence.

### 3.1 Kinematic evidence

Although direct motion-capture evidence from pianist–dancer interaction remains limited, adjacent coordination paradigms are informative when they preserve the same functional problem structure, namely the anticipatory extraction of visible kinematic information under tight temporal constraints. In piano duets, pianists flexibly use nonverbal visual cues (in addition to auditory cues) to maintain coordination, especially in contexts where timing is underspecified or unstable (Bishop and Goebl, 2015). Moreover, motion-based analyses show that ensemble musicians' cueing gestures communicate beat position and tempo, offering quantifiable kinematic signals that can support anticipatory alignment (Bishop and Goebl, 2018a). Gesture kinematics and conventionality also predict synchronization success in piano duos, consistent with a cue-based predictive control account (Bishop and Goebl, 2018a).

### 3.2 Neurophysiological evidence

Neurophysiological evidence for “seeing sound” is currently clearer in adjacent musician-observation paradigms than in pianist–dancer studies *per se*; accordingly, the studies reviewed here are used to constrain plausible mechanisms rather than to claim direct neural evidence from pianist–dancer dyads themselves. In professional pianists, observing piano-playing actions engages transmodal sensorimotor networks consistent with internal simulation of action–sound mappings (Haslinger et al., 2005), and converging fMRI evidence indicates partially shared networks for auditory and motor processing in expert pianists (Bangert and Altenmüller, 2006). In the dance domain, naturalistic EEG work further shows that dance or music expertise modulates spectators' cortical oscillations while watching live dance with music, consistent with expertise-dependent predictive processing during movement observation (Poikonen et al., 2024). Importantly, however, musicians' advantages in visual rhythm discrimination are not always explained by direct cross-modal recruitment of auditory cortex, suggesting that higher-level multimodal mechanisms may be involved (Korczyk et al., 2022).

## 4 Hearing movement: empirical evidence for auditory–motor planning

In audiovisual coordination in dance, dancers do not rely on visual cues alone. They also use auditory feedback to adjust their movement trajectories. With fine grained auditory perception, dancers can synchronize movement with musical rhythm, respond to micro timing perturbations, and regulate bodily state. This section synthesizes behavioral and physiological studies to show how dancers use auditory information to guide motor planning.

### 4.1 Behavioral evidence: micro-timing and movement alignment

Sensorimotor synchronization (SMS) refers to the process by which movers align periodic actions (e.g., tapping or stepping) with an auditory beat. In paced tapping paradigms, small and unexpected timing perturbations reliably elicit rapid phase correction, including asymmetric responses to advances vs. delays, indicating predictive error correction rather than purely reactive control (Repp, 2001; Repp and Su, 2013). More recent work further shows that the *perturbation context* (the distribution/type of perturbations experienced) can recalibrate the error-correction mechanism and reshape the resynchronization curve, including the degree of response asymmetry (Silva and Laje, 2024). Extending beyond finger tapping, gait/stepping studies demonstrate that adapting footfall timing to auditory perturbations affects the stability of ongoing walking, consistent with continuous predictive updating in music–movement alignment (Ravi et al., 2021).

### 4.2 Physiological evidence: auditory cues and autonomic drive

Auditory structure can modulate movers' autonomic state in ways relevant to motor readiness. For example, music-evoked tension (e.g., delayed harmonic resolution) has been validated using skin conductance response (SCR), with tension-evoking excerpts reliably associated with heightened SCR alongside increased subjective tension ratings (Kurzom and Mendelsohn, 2022). In addition, rhythm structure and task demands (perception vs. synchronization) can shape cardiac dynamics, suggesting that auditory temporal complexity can couple to autonomic regulation during timing tasks (Wright and Palmer, 2023). Together, these findings support the view that auditory cues tune not only overt timing correction but also physiological readiness during music–movement coordination.

## 5 Closed-loop coordination, mathematical and hyperscanning evidence

In cross-modal joint performance between a pianist and a dancer, coordination is not a one-way audiovisual response. It is a closed-loop system grounded in deep behavioral and neural coupling. This section synthesizes empirical evidence from coupled oscillator modeling and EEG hyperscanning to explain how partners overcome inherent neural delays and achieve bidirectional synchrony and dynamic coupling.

### 5.1 Computational modeling, coupled oscillator parameters

Coupled-oscillator models provide a standard mathematical framework for quantifying synchronization in multi-agent systems. In music–dance interaction, the pianist's and dancer's rhythms can be idealized as interacting oscillatory processes whose dynamics are governed by coupling strength and phase dynamics (Haken et al., 1985; Kelso, 1995). Within this modeling family, coupling parameters can be interpreted as quantifying how strongly partners mutually attract/adjust, and how asymmetries in action–perception coupling map onto different interpersonal synchronization strategies (e.g., mutual adaptation, leading–following, leading–leading; Heggli et al., 2019, 2021). Consistent with this framework, leader–follower dynamics in synchronized interaction have been linked to asymmetric neural signatures in dual-EEG paradigms, supporting the relevance of asymmetric coupling in interpersonal coordination (Konvalinka et al., 2014).

### 5.2 Inter-brain synchrony, hyperscanning and dyadic neural evidence

EEG hyperscanning and related “two-person neuroscience” methods allow direct tests of whether interpersonal coordination is supported by cross-brain coupling in addition to within-brain action–perception processes. Inter-brain synchronization has been reported in controlled joint-action paradigms such as imitation (Dumas et al., 2010) and musical duets (Sänger et al., 2012). For dance-relevant contexts, dyadic EEG work further indicates that neural signals can track and predict coordination-relevant features of social interaction during coupled dance (e.g., visual-contact–related effects; Bigand et al., 2025), while computer-vision approaches quantify interpersonal synchrony and interaction quality directly from movement kinematics in joint dance (Grinspun Siguelnitzky et al., 2025). At the same time, interpretation of inter-brain metrics requires caution because shared sensory input, movement artifacts, and analytic choices can inflate apparent coupling; recent methodological syntheses emphasize combining behavioral coupling models with rigorous preprocessing and appropriate null models (Hakim et al., 2023; Hamilton, 2021; Zamm et al., 2024).

## 6 Discussion

### 6.1 Integrative synthesis

Taken together, the reviewed evidence supports a cautiously integrative account of pianist–dancer coordination (Abalde et al., 2024; De Vicariis et al., 2024; Vuust et al., 2022). Across adjacent kinematic, behavioral, and computational literatures, the most consistent pattern is that coordination is unlikely to depend on late, purely reactive feedback alone. Instead, performers appear to rely on anticipatory use of multimodal cues, ongoing error correction, and bidirectional adaptation under uncertainty (Abalde et al., 2024; De Vicariis et al., 2024; Heggli et al., 2019, 2021; Vuust et al., 2022). Kinematic studies suggest that visible preparatory movement can function as advance information for timing alignment (Bishop and Goebl, 2015, 2018a,b; Wakiyama et al., 2025); sensorimotor-synchronization work indicates that auditory perturbations elicit rapid predictive correction rather than simple delayed reaction (Ravi et al., 2021; Repp, 2001; Repp and Su, 2013; Silva and Laje, 2024); and coupled-oscillator and hyperscanning approaches converge on the idea that interpersonal coordination is dynamically co-regulated rather than unidirectionally driven (Bigand et al., 2025; Dumas et al., 2010; Heggli et al., 2019, 2021; Sänger et al., 2012; Zamm et al., 2024). What can be said with confidence, therefore, is not that pianist–dancer interaction has already been definitively explained by predictive coding, but that an inference-based, cross-modal account currently offers a coherent way to organize the available evidence and generate testable predictions for more direct dyadic research (Abalde et al., 2024; De Vicariis et al., 2024; Vuust et al., 2022).

### 6.2 Limitations

Current evidence remains limited in three ways. First, most studies use laboratory or semi-naturalistic tasks (e.g., fixed excerpts, metronomes, or single-modality manipulations), and generalization to highly coupled on-stage performance is uncertain. Second, multimodal synchronized acquisition (motion capture, audio, eye tracking, and EEG) requires stringent timestamp alignment and artifact suppression; in practice, mobile/field EEG often relies on low-cost, low-channel systems and is vulnerable to movement-related noise, which can reduce interpretability (García-Monge et al., 2024), while recent mobile neuroscience platforms highlight the engineering complexity of multi-sensor synchronization in real environments (Amaro et al., 2025). Third, hyperscanning provides many inter-brain coupling metrics, yet cross-study comparability and causal interpretation remain contested (Hakim et al., 2023; Zamm et al., 2024).

### 6.3 Future directions

Future work can advance along two directions. First, portable EEG hyperscanning should be deployed in real rehearsals and performances—paired with rigorous synchronization frameworks and fine-grained event annotation—to test whether fronto-parietal phase locking covaries with behavioral correction in high-difficulty passages (Zamm et al., 2024). Second, embodied AI accompaniment and other live music agents can combine computational synchronization models (e.g., coupled-oscillator controllers) with legible non-sounding gestures to improve predictability and coordination in human–machine performance (Gao et al., 2024; Heggli et al., 2019; Kim et al., 2026).
